# A Cavity-Tailored Metal-Organic Tetrahedral Nanocage and Gas Adsorption Property

**DOI:** 10.3390/nano12244402

**Published:** 2022-12-09

**Authors:** Xin Jin, Hui Jiang, Yi Chen, Xin Han, Ken Sun, Linlin Shi, Xin-Qi Hao, Mao-Ping Song

**Affiliations:** 1 College of Chemistry, Zhengzhou University, Zhengzhou 450001, China; 2 College of Energy and Power Engineering, North China University of Water Resources and Electric Power, Zhengzhou 450045, China

**Keywords:** porous organic metal nanocages, self-assembly, adsorbent, gas separation and storage

## Abstract

Porous organometallic nanomaterials are a new class of materials based on a three-dimensional structure. They have excellent applications in different fields, but their applications in gas storage and separation have not been fully developed. CO_2_ adsorption storage and hydrocarbon separation has been a challenging industrial problem. Several typical molecular adsorbents have been used to study the separation, but the problems of long-term stability, high selectivity and synthetic complexity of these adsorbents remain to be solved. Here, we have designed and synthesized tetrahedral metal supramolecular nanocage with custom cavities based on the unique rigid structure of triptycene derivatives. Using the unique discrete porous structure of tetrahedral metal nanocages, the gas adsorption and separation performance of the metal supramolecular nanocage was investigated. By analyzing the adsorption and desorption isotherms and the multi-component competitive adsorption curves, we noticed that the tetrahedral supramolecular nanocages had good CO_2_ storage capacity and good separation capacity for C_2_H_2_/CO_2_ and C_2_H_2_/N_2_. All these indicate that porous organic metal nanomaterials are expected to be a new energy saving separation material.

## 1. Introduction

A variety of supramolecules with attractive structures and flexible properties have been successfully generated by self-assembly strategy [1,2,3,4]. Metal–organic supramolecular cages (MOCs) are discrete molecules constructed by coordination-driven self-assembly of organic connectomes and metal ions or clusters [5,6]. They has become one of the research hotspots in the past two decades. Based on their unique multifunctional topology, metal–organic supramolecular cages have been used in many fields, including catalysis [7,8,9,10], molecular recognition [11,12,13,14,15], biological simulation [16,17,18,19], guest isolation [20,21], reactive species stability [22,23], drug delivery [24,25], membrane transport [26,27], etc. However, MOCs have not been fully developed as adsorbents, especially for hydrocarbon storage and separation.

In recent years, the excessive carbon dioxide produced by the widespread use of fossil fuels has become the main culprit of the greenhouse effect. Acetylene as a basic feedstock for various industrial materials is also a key fuel [28], mainly produced through the cracking of hydrocarbons or partial combustion of methane [29,30], of which carbon dioxide is the main impurity. Up to date, the commonly used C_2_H_2_/CO_2_ separation technology usually relies on low temperature rectification or liquid absorbent, which has high cost and low efficiency [31]. Therefore, there is a great demand for the development of alternative and energy-saving separation technologies [32,33]. Currently, non-thermally driven approaches such as adsorption or membrane separation have received a lot of attention due to their lower energy penalties. However, the adsorption separation performance depends largely on the nature of the adsorbent. In the past two decades, many microporous materials (MPM) for gas storage and separation have been studied, such as polymer membranes [34], inorganic porous materials [35,36], graphene [37], metal–organic frameworks (MOFs) [38,39], covalent organic frameworks (COFs) [40,41], hydrogen-bonded organic frameworks (HOFs) [42,43], etc. However, they lack long-term stability [33,44], and only a minority of them have high selectivity [45,46,47]; thus, it is necessary to develop new molecular and supramolecular adsorbents with good stability and high selectivity. It is known that many discrete porous metal–organic nanocages (MOCs) have topological diversity to ensures higher BET surface area and larger adsorption capacity [48], adjustable size cavities to improve selectivity, and their easy preparation characteristics [49]. Given this, they are expected to be the next generation of adsorbents for energy efficient separation.

Tetrahedral metal supramolecular nanocage molecular adsorbents with 3D structures have shown excellent performance in many discrete porous metal organic nanocages (MOCs), such as the separation of methane, ethane and ethylene [50]. These kinds of supramolecular nanocage have attracted much attention due to their unique customizable cavities, high porosity and easy preparation. In this work, we purposefully designed and constructed tetrahedral nanocage **Cage 1** with triptycene derivatives as faces and metal cations as vertices by coordination driven collaborative self-assembly. Based on the unique three-dimensional rigid structure of triptycene derivatives and their unique electron-rich cavities, which are beneficial to improve the gas adsorption capacity and selectivity, the CO_2_ storage capacity and hydrocarbon separation ability of **Cage 1** were tested. Not surprisingly, the gas adsorption experiments showed that the adsorption capacities of **Cage 1** for CO_2_, O_2_, N_2_ and C_2_H_2_ at 298 K and 1 bar, respectively, were 5.1711 cm^3^g^−1^, 0.4806 cm^3^g^−1^, 0.5684 cm^3^g^−1^ and 10.8463 cm^3^g^−1^. **Cage1** has obvious differential adsorption capacity for different gases and high adsorption capacity for CO_2_. This makes **Cage 1** possible as a new nano adsorbent for CO_2_ storage and hydrocarbon gas separation.

## 2. Materials and Methods

### 2.1. General Information

All the chemical reagents were purchased from commercial sources and directly used without further purification. Reactions were monitored by Thin Layer Chromatography (TLC) using UV light (254/365 nm). Products were purified by column chromatography, which was carried out on 200–300 mesh of silica gel. NMR spectra data were recorded on a 600 MHz Bruker NMR spectrometer, Germany. The UV-Vis experiments were conducted on dual-beam UV-Vis spectrophotometer (TU-1901), PERSEE, Beijing, China. The fluorescent experiments were recorded on Horiba-FluoroMax-4 spectrofluorometer, HORIBA, Edison, NJ, USA. The high-pressure fluorescence spectra under hydrostatic conditions were measured using a fluorescence microscope (IX71, Olympus, 50, NA = 0.5, Japan) equipped with a spectrometer (Jobin Yvon iHR320, USA). The light source was a mercury lamp with an excitation wavelength of 365 nm.

ESI-MS was recorded with a Waters Synapt G2 -Si mass spectrometer, USA; using solutions of 0.01 mg sample in 1 mL of CHCl_3_/CH_3_OH (1:3, *v*/*v*) for ligands or 0.5 mg sample in 1 mL of CH_3_CN/CH_3_OH (3:1, *v*/*v*) for supramolecules. High-resolution electrospray ionization mass spectrometry (HR-ESI MS) experiments were performed with a Water Q-Tof Micro MS/MS high resolution mass spectrometer in ESI mode.

Thermogravimetric analyses (TGA) were carried out on a Netzsch STA 449 F5 thermal analyzer, Germany; under a nitrogen atmosphere with a heating rate of 10 °C·min^−1^.

Low-pressure gas adsorption measurements were performed on a Micromeritics Instrument Corporation TriStar II 3020 Version 3.02 surface area analyze, USA; Samples were degassed under dynamic vacuum for 12 h at 60 °C prior to each measurement. N_2_ isotherms were measured using a liquid nitrogen bath (77 K).

All the gas adsorption–desorption measurements were carried out by using an automatic volumetric adsorption equipment (Micromeritics, ASA 2460), USA; at 298 K. Prior to the measurements, the samples were degassed at 120 °C under dynamic vacuum for 10 h to remove the adsorbed impurities.

### 2.2. General Experimental Procedures for the Synthesis of L and Cage 1

Synthesis of **L**:

Compounds **1**, **2**, **3** and **4** were synthesized according to published procedures [51].

4-aminophenylboronic acid pinacol ester (289.08 mg, 1.32 mmol), compound **4** (196.40 mg, 0.4 mmol), sodium carbonate (127.2 mg, 1.2 mmol) and Pd(PPh_3_)_4_ (69.30 mg, 0.06 mmol) were added to a round bottom flask containing DMF/H_2_O (18 mL/6 mL) under nitrogen atmosphere and reacted at 110 °C for 16 h. Then, the mixture was cooled to room temperature, extracted with ethyl acetate (200 mL × 3). The combined organic phase was washed with brine (200 mL × 3), dried over Na_2_SO_4_ and the solvent was evaporated. The crude product was purified on a silica gel column using petroleum and ethyl acetate (1:1) as the eluent to obtain pale yellow solid **L** (84.35 mg, 40%). ^1^H NMR (600 MHz, CDCl_3_) δ 7.57 (s, 3H), 7.40 (d, J = 7.6 Hz, 3H), 7.31 (d, J = 8.3 Hz, 6H), 7.14 (d, J = 7.6 Hz, 3H), 6.68 (d, J = 8.3 Hz, 6H), 5.48 (d, J = 9.8 Hz, 2H), 3.61 (s, 6H); ^13^C NMR (151 MHz, CDCl_3_) δ 145.8, 145.7, 145.6, 143.3, 143.3, 138.7, 138.6, 131.8, 128.1, 123.8, 123.8, 123.3, 123.3, 122.1, 122.0, 115.3, 54.1, 53.6. HR-ESI MS (m/z) calcd for C_38_H_29_N_3_^+^ [M+H]^+^: 528.2434; found: 528.2433.

Synthesis of **Cage 1**:

Zn(NTf_2_)_2_ (9.38 mg, 15.00 µmol, 1.5 equiv), L (5.23 mg, 10.00 µmol, 1 equiv) and pyridyl aldehyde (3.43 mg, 32.00 µmol, 3.2 equiv) were added to a reaction tube, then 1.5 mL acetonitrile was added. The whole reaction mixture was reacted at room temperature for 8 h. Ethyl ether (30 mL) was added into the reaction mixture. The yellow solid was collected by centrifugation and dried under reduced pressure, **Cage 1** was obtained by quantitative yield. ESI-MS (*m*/*z*): Calcd. [C_224_H_144_N_24_Zn_4_–8NTf_2_^−^]8^+^ for: 430.13, found: 430.14. Calcd. [C_226_H_145_F_6_N_25_O_4_S_2_Zn_4_–7NTf_2_^−^]7^+^ for: 531.56, found: 531.56. Calcd. [C_228_H_146_F_12_N_26_O_8_S_4_Zn_4_–6NTf_2_^−^]6^+^ for: 666.80, found: 666.79. Calcd. [C_230_H_147_F_18_N_27_O_12_S_6_Zn_4_–5NTf_2_^−^]5^+^ for: 856.35, found: 856.30. Calcd. [C_232_H_148_F_24_N_28_O_16_S_8_Zn_4_–4NTf_2_^−^]4^+^ for: 1140.41, found: 1140.54. Calcd. [C_234_H_149_F_30_N_29_O_20_S_10_Zn_4_–3NTf_2_^−^]3^+^ for: 1613.86, found: 1613.80.

## 3. Results and Discussion

**Scheme 1:**
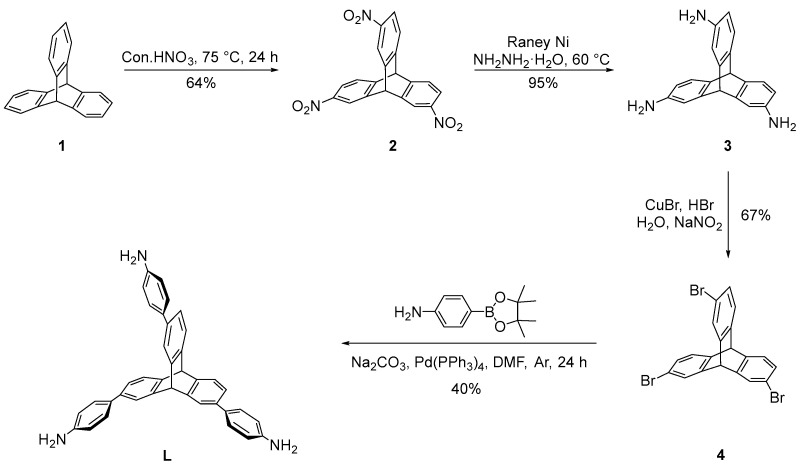
Synthesis of **L**.

Triptycene-based **L** was obtained in four steps from commercially-available starting materials (Scheme 1). According to procedures previously reported, using triptycene as the starting material. At first, triptycene was treated with nitric acid under reflux conditions to give **2** in 64% yield. Next, the compound **2** derivatives were reduced by Raney-Ni in the presence of hydrazine hydrate to yield the **3** in quantitative yield. Then, compound **3** diazotized with NaNO_2_ was added under reflux conditions of HBr and CuBr to give **4** in 67% yield. Finally, under the conditions of 110 °C reaction temperature, tetrakis(triphenylphosphine)palladium as catalyst, sodium carbonate as base, DMF and H_2_O as the reaction solvent, the Suzuki reaction with 4-amnophenylboronic acid pinacol ester was carried out to obtain **L** with 40% yield.

**Figure 1:**
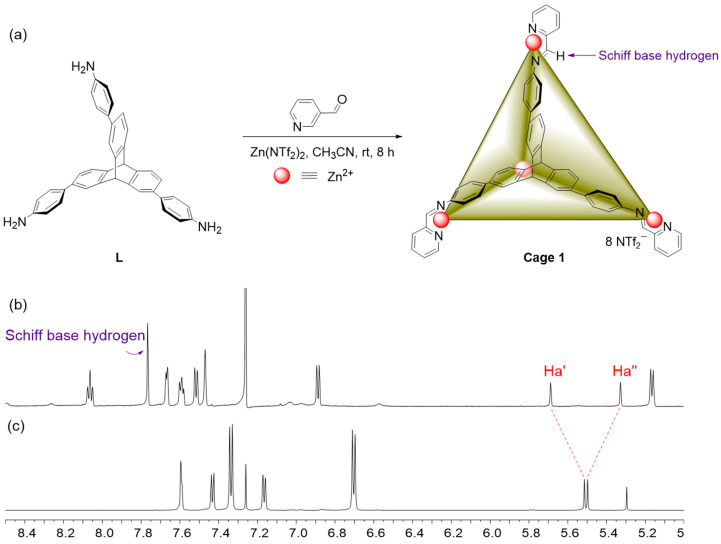
(**a**) Coordination-driven self-assembly and geometric structure of **Cage 1** (ZnII_4_L_4_). ^1^H NMR spectra (600 MHZ, CD_3_CN/CDCl_3_, 298 K) of **Cage 1** (**b**) and **L** (**c**).

With the composition of **L**, the reaction of **L** (4 equiv) with Zinc bis(trifluoromethylsulfonyl)imide (4 equiv) and 2-formylpyridine (12 equiv) in acetonitrile at room temperature for 8 h, affording desired tetrahedral **Cage 1** as a yellow solid in a quantitative yield (Figure 1a). 

As shown in Figure 1b,c, ^1^H NMR spectrum of **Cage 1** was thoroughly different from its assembled precursors, implying the formation of a mew substance. Depending on previous literature reports, we supposed that the **Cage 1** should be a regular tetrahedral nanostructure and simultaneously has a certain size cavity. Fortunately, the aforementioned speculation was also demonstrated by the ^1^H NMR and ^13^C. Through ^1^H NMR contrast of **Cage 1** and **L** (Figure 1), the resonances of the protons on the center of triptycene of ligand **A** were equally divided into two sets of different signal peaks after the formation of **Cage 1**. One set (Ha′) appeared at lower chemical shift and the other set (Ha′′) at higher field shift as a result of the strong shielding effect of the **Cage 1**. The regular splitting of the group of protons shows that half of the two hydrogens in the center of triptycene were located outside the cage and the other half of them were encapsulated inside the cavity of **Cage 1**. In the same breath, the hydrogen on the pyridine formaldehyde also disappeared with the synthesis of the cage, which proves the formation of the tetrahedral metal supramolecular nanocage.

**Figure 2:**
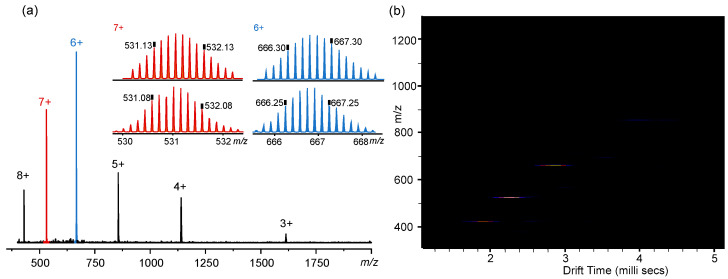
(**a**) ESI-MS spectra of **Cage 1**. (**b**) ion mobility profiles of **Cage 1**.

Electrospray ionization mass spectrometry (ESI-MS) date demonstrated that **Cage 1** are substantially stable in solution. Furthermore, the molecular weight of **Cage 1** was accurately determined and the tetrahedral structure of **Cage 1** was confirmed. (See the Supporting Information for details). As shown in Figure 2a, the ESI-MS of **Cage 1** successively revealed a set of dominant peaks with persistent charge states ranging from. 3+ to 8+ with the sequential loss of NTf_2_^−^ (Figure S5 see the Supporting Information for details). Experimental isotope patterns for each peak matched faultless with simulated isotope patterns, indicative of the formation of a supramolecular ensemble with the proposed stoichiometry. After deconvolution, the average molar masses of self-assembled **Cage 1** were 5672 Da, respectively, matching faultless with its expected chemical composition [(C_56_H_38_N_6_)_4_Zn_4_(NTf_2_)_8_]. In addition, ion mobility profiles in Figure 2b shows that **Cage 1** is a single compound, ruling out the possibilities of forming other undesired complexes and isomers.

**Figure 3:**
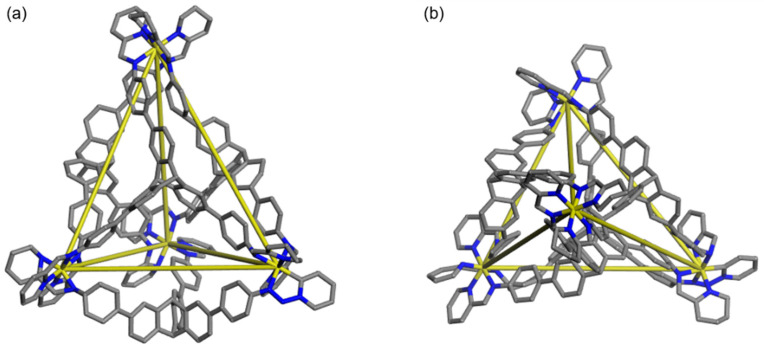
Energy-minimized molecular model of **Cage 1**. (Zn, yellow; N, blue; C, gray). Hydrogen atoms and counter anions are omitted for clarity: (**a**) side view and (**b**) top view.

Numerous efforts have been made to obtain complete data for single crystal X-ray analysis were unsuccessful. Therefore, we constructed the simulated molecular model of **Cage 1** (Figure 3 and Figure S7 see the Supporting Information for details). Simulated molecular models show that the supramolecular nanostructure of **Cage 1** perfectly matches the designed tetrahedron. The two protons at the center of the triptycene are divided into two groups, with one group pointing inside the cavity and the other group pointing outside. The distance between two adjacent zinc ions in the tetrahedron is 17.4 Å as measured by the Materials Studio software.

Triptycene has a unique three-dimensional rigid structure, the included angle between the three benzene rings is 120 degrees, forming three open negative electron cavities. Consequently, after **L** generates **Cage 1** through self-assembly, its internal cavity is electronegative, which is more conducive to encapsulating positively charged molecules. Taking into consideration the unique structure of **Cage 1**, we envisaged this space to be suitable for the binding of small hydrocarbon gases via van der Waals interactions and can be resolved by the independent cavity size of the **Cage 1**. Hence, its gas adsorption behavior was studied. First, **Cage 1** was obtained as a powder following precipitation of **Cage 1** from acetonitrile by adding diethyl ether. The porous features of **Cage 1** prompted us to examine their stability and gas uptake behavior. Thermogravimetric analysis (TGA) of **Cage 1** shows a 4% weight loss in the temperature range of 0–100 °C under N_2_, corresponding to the loss of water molecules and the framework is stable up to ca. 400 °C (Figure S8 see the Supporting Information for details). **Cage 1** was thus activated by heating at 120 °C under vacuum (<0.013 mBr) for 10 h to remove solvent. Powder X-ray diffraction (PXRD) of **Cage 1** revealed no distinct sharp peaks, consistent with the presence of amorphous material (Figure S7 see the Supporting Information for details).

**Figure 4:**
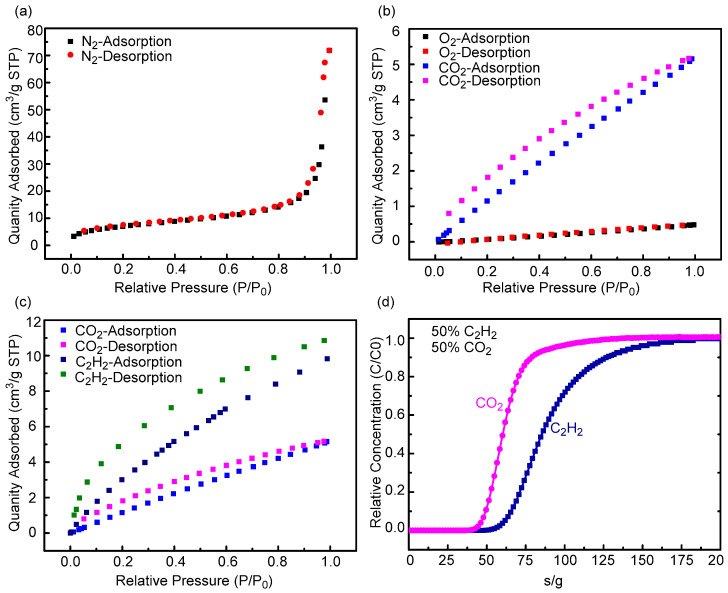
(**a**) N_2_ adsorption and desorption isotherms of **Cage 1** at 298 K. (**b**) CO_2_ and O_2_ adsorption and desorption isotherms of **Cage 1** at 298 K. (**c**) C_2_H_2_ and CO_2_ adsorption and desorption isotherms of **Cage 1** at 298 K. (**d**) Transient breakthrough simulation for separation of equimolar C_2_H_2_/CO_2_ mixture using **Cage 1** at 298 K, with a partial pressure of 50 KPa for each.

By analyzing the N_2_ adsorption and desorption curve of the **Cage 1** at 77 K, we can obtain the information of its aperture structure. As can be seen from Figure 4, the N_2_ isotherm of **Cage 1** shows a reversible type-II curve with point B at low P/P_0_. It can also be seen from Figure 4a that **Cage 1** adsorption is lower at 77 K and lower pressure, and the adsorption capacity of N_2_ increases with the increasing of the pressure. When P/P_0_ is approach 1, the adsorption value reaches the maximum, the maximum adsorption capacity of N_2_ by **Cage 1** is 71.85397 cm^3^g^−1^ at 77 K and 1 bar, and its BET surface area is 25.3955 m^2^g^−1^ (Figure S9 see the Supporting Information for details). To sum up, these data suggest that both micropores and mesoporous exist in **Cage 1**, and this is consistent with the regular tetrahedral nanopore structure of **Cage 1**.

Furthermore, we also examined the gas adsorption behaviors of **Cage 1** for CO_2_ and O_2_. According to the isothermal curve of CO_2_ and O_2_ of **Cage 1** at 298 K as shown in Figure 4b, it can be procured that the adsorption and desorption of CO_2_ are positively correlated with the pressure. It maximizes at P/P_0_ = 1, the amount of adsorption and desorption is 5.1711 cm^3^g^−1^. The adsorption capacity and desorption capacity of O_2_ are similar to CO_2_, and also positively correlated with pressure. When P/P_0_ = 1, the maximum adsorption capacity is 0.4806 cm^3^g^−1^. The adsorption behavior of **Cage 1** for CO_2_ and O_2_ is physical adsorption. Hence, the different adsorption capacity of **Cage 1** for CO_2_ and O_2_ is mainly due to the different molecular weight and structure of CO_2_ and O_2._ We believe that the size of O_2_ is relatively small and unable match the cavity size of **Cage1**; hence, it cannot be adsorbed by the cavity, while CO_2_ can be adsorbed by the Cage cavity due to its larger linear spatial structure and relatively high matching degree with the Cage cavity. Beyond that, the hysteresis loop in the adsorption and desorption curve shown in the Figure 4b may be caused by capillary condensation hysteresis, which is mainly affected by the aperture and structure of the cage itself, and has nothing to do with the adsorbent.

Acetylene (C_2_H_2_) is an essential chemical feedstock for modern petrochemical products such as acrylic acid derivatives, vinyl chloride, plastics and rubber. Acetylene is mainly produced via natural gas combustion or hydrocarbon. However, CO_2_ as an impurity always exists in the production process of acetylene, due to their highly similar molecular size, shape, boiling points (boiling points: C_2_H_2_, 189.3 K; CO_2_, 194.7 K; molecular shapes and size: C_2_H_2_, 3.3 × 3.3 × 5.7 Å^3^; CO_2_, 3.2 × 3.3 × 5.4 Å^3^) and almost identical kinetic diameters of ~ 3.3 Å^3^ [52,53]. Hence, how to efficiently separate acetylene and carbon dioxide has been a problem that unable be ignored in industrial production of acetylene. The isothermal curve of **Cage 1** to C_2_H_2_ of desorption in Figure 4c was analyzed. We found that the adsorption capacity of the **Cage 1** to C_2_H_2_ was similar to that of CO_2_; all were positively correlated with pressure. When P/P_0_ = 1, the maximum adsorption capacity is 10.8463 cm^3^g^−1^. By comparison, the **Cage 1** adsorbed about twice as much to C_2_H_2_ as it did to CO_2_. Hence, we think that **Cage 1** might have the property of separating C_2_H_2_ and CO_2_. To further evaluate the actual separation performance of the C_2_H_2_/CO_2_ mixture in **Cage 1**, laboratory scale fixed-bed breakthrough experiments were carried out under condition of C_2_H_2_/CO_2_ (50/50) gas mixture. As Figure 4d shows that **Cage 1** achieves effective separation of C_2_H_2_/CO_2_ within a certain range. It can be observed from Figure 4d that CO_2_ was first eluted and then rapidly approached a pure grade without detectable C_2_H_2_, then C_2_H_2_ is gradually eluted. The only fly in the ointment is that C_2_H_2_ stays in the packed column for a short time, which leads to the inefficient separation of C_2_H_2_/CO_2_ in the **Cage 1**. This is related to the structure of the **Cage 1** itself and the size of the aperture. In addition, the loading level of **Cage 1** samples in the packed column is low, so that the saturation absorption value of C_2_H_2_ is quickly reached, which also has a non-negligible impact on the separation efficiency.

**Figure 5:**
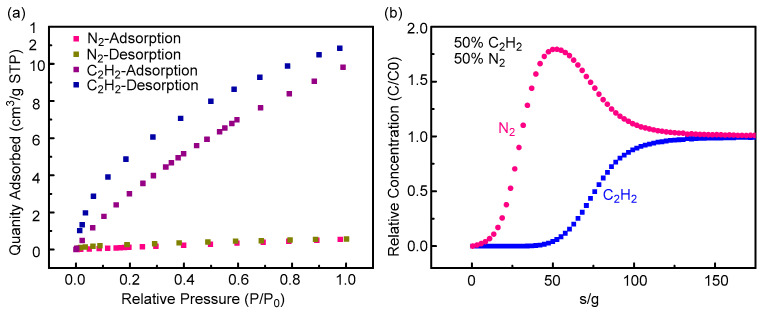
(**a**) C_2_H_2_ and N_2_ adsorption and desorption isotherms of **Cage 1** at 298 K. (**b**) Transient breakthrough simulation for separation of equimolar C_2_H_2_/N_2_ mixture using **Cage 1** at 298 K, with a partial pressure of 50 KPa for each.

The calcium carbide process is a common process to produce acetylene. When calcium carbide is added, nitrogen replacement must be carried out on the feeding storage hopper of acetylene generator to prevent explosion. Then, a N_2_ /C_2_H_2_ mixture will be discharged in this process. This leads to waste of N_2_ and C_2_H_2_ and pollution of the environment. Hence, it is of great significance to effectively separate and recover the emissions N_2_ and C_2_H_2_. According to the analysis of the isotherms of the **Cage 1** for C_2_H_2_ and N_2_ at 298 K in Figure 5a, the adsorption capacity of the **Cage 1** for acetylene and nitrogen all increases with the increase in pressure. When P/P_0_ = 1, the adsorption capacity of the **Cage 1** for N_2_ reaches a maximum of 0.5684 cm^3^g^−1^. By comparison, it was found that the adsorption capacity of **Cage 1** for acetylene was about 20 times that of **Cage 1** for nitrogen. Therefore, in order to further prove that **Cage 1** demonstrates good separation performance for C_2_H_2_ and N_2_, experiments were carried out under dynamic instantaneous breakthrough conditions to further evaluate the actual separation performance of **Cage 1** in C_2_H_2_/N_2_ gas mixture. In these experiments, the gas mixture with 2 mL/min was the total flow through the packed tower. According to the C_2_H_2_/N_2_ (50/50) penetration curve in Figure 5b, **Cage 1** can efficiently separate the C_2_H_2_/N_2_ mixture. As shown in the figure, N_2_ was eluted first because **Cage 1** has a small adsorption capacity for N_2_. At this time, pure Nitrogen productivities, calculated as an area under the breakthrough curves, for the equimolar gas mixtures were 7.6 mL (STP) g^−1^ (0.3 mol kg^−1^) for N_2_/C_2_H_2_. (Figure S14 see the Supporting Information for details). Then C_2_H_2_ is retained in the packed column for a long time until the saturation adsorption capacity is broken. Therefore, the purpose of efficient separation of acetylene and nitrogen is achieved.

## 4. Conclusions

In conclusion, we have successfully constructed triptycene-functionalized supramolecular metal–organic tetrahedral cages through coordination-driven synergetic self-assembly, which were thoroughly characterized by NMR and ESI-MS. Considering the unique three-dimensional rigid structure and high porosity of triptycene derivatives, we investigated the gas adsorption properties of the **Cage 1**. Through a large amount of data, we found some interesting, but not surprising, phenomenon. **Cage 1** has a good adsorption ability for some hydrocarbon gas molecules, such as C_2_H_2_, leading also to a high adsorption ability of the main culprit of greenhouse effects, CO_2_; however, on small molecules, such as O_2_ and N_2_, it has almost no adsorption capacity, which is beneficial to the separation of CO_2_ and O_2_. The remarkable thing is that **Cage 1** also has good separation performance for C_2_H_2_/CO_2_ and C_2_H_2_/N_2_. It has a good application prospect in the industrial production and separation of acetylene. Unlike some complex synthesis methods of porous coordination polymers (PCP) or metal-organic frameworks (MOFs) previously reported, **Cage 1** is easier to obtain, can be prepared in gram scale, and has good stability at room temperature. This study provides a new way to solve the difficult problem of CO_2_ storage and the difficult separation of C_2_H_2_/CO_2_ and C_2_H_2_/N_2_ in industrial production, and also provides a possibility to develop a new porous metal–organic nanocage adsorbents that can be prepared and used in an industrial scale, which has broad application prospects.

## Data Availability

Not applicable.

## Supplementary Materials

The following supporting information can be downloaded at: https://www.mdpi.com/article/10.3390/nano12244402/s1. Figure S1: ^1^H NMR (600 MHZ, CDCl_3_) spectrum of **L**.; Figure S2: ^13^C NMR (151 MHZ, CDCl_3_) spectrum of **L**.; Figure S3: ^13^C NMR (151 MHZ, CDCl_3_/CD_3_CN 1:1) spectrum of **L**.; Figure S4: ^1^H NMR (151 MHZ, CDCl_3_/CD_3_CN 1:1) spectrum of **Cage 1**.; Figure S5: (a) and (b) ESI-MS spectrum of **Cage 1**.; Theoretical (top) and experimental (bottom) isotope patterns for different charge states observed from **Cage 1** (NTf_2_^−^ as the counterion).; Figure S6: Energy-minimized molecular model of **Cage 1**. (Zn, yellow; N, blue; C, gray). Hydrogens, counter anions are omitted for clarity.; Figure S7: Experimental PXRD pattern of **Cage 1**.; Figure S8: TGA plot of solid **Cage 1** under N_2_.; Figure S9: N_2_ sorption isotherms of activated **Cage 1** measured at 77 K (upper) and plot of the linear region of the BET equation for **Cage 1** (bottom).; Figure S10: CO_2_ adsorption/desorption isotherms of activated **Cage 1** at 298 K.; Figure S11: O_2_ adsorption/desorption isotherms of activated **Cage 1** at 298 K.; Figure S12: C_2_H_2_ adsorption/desorption isotherms of activated **Cage 1** at 298 K.; Figure S13: N_2_ adsorption/desorption isotherms of activated **Cage 1** at 298 K.
