# Supplementary material for: A Cavity-Tailored Metal-Organic Tetrahedral Nanocage and Gas Adsorption Property
Source: Nanomaterials (Basel). 2022 Dec 9;12(24):4402. doi: 10.3390/nano12244402 (PMC9783787; doi:10.3390/nano12244402)
Supplement: Supplementary file 1 [file nanomaterials-12-04402-s001.zip › nanomaterials-2033777-supplementary.pdf]

Supplementary Materials

A Cavity-Tailored Metal-Organic Tetrahedral Nanocage and Gas Adsorption Property

Xin Jin [1,†], Hui Jiang [1,†], Yi Chen [1], Xin Han [1], Ken Sun [1,2,*], Linlin Shi [1,*], Xin-Qi Hao [1,*] and Mao-Ping Song [1,*]

[1] College of Chemistry, Zhengzhou University, Zhengzhou 450001, China
[2] College of Energy and Power Engineering, North China University of Water Resources and Electric Power, Zhengzhou 450045, China
* Correspondence: sunken0371@126.com (K.S.); linlinshi@zzu.edu.cn (L.S.); xqhao@zzu.edu.cn (X.-Q.H.);
† These authors equally contributed to this work.

1. The characterization of L, Cage 1

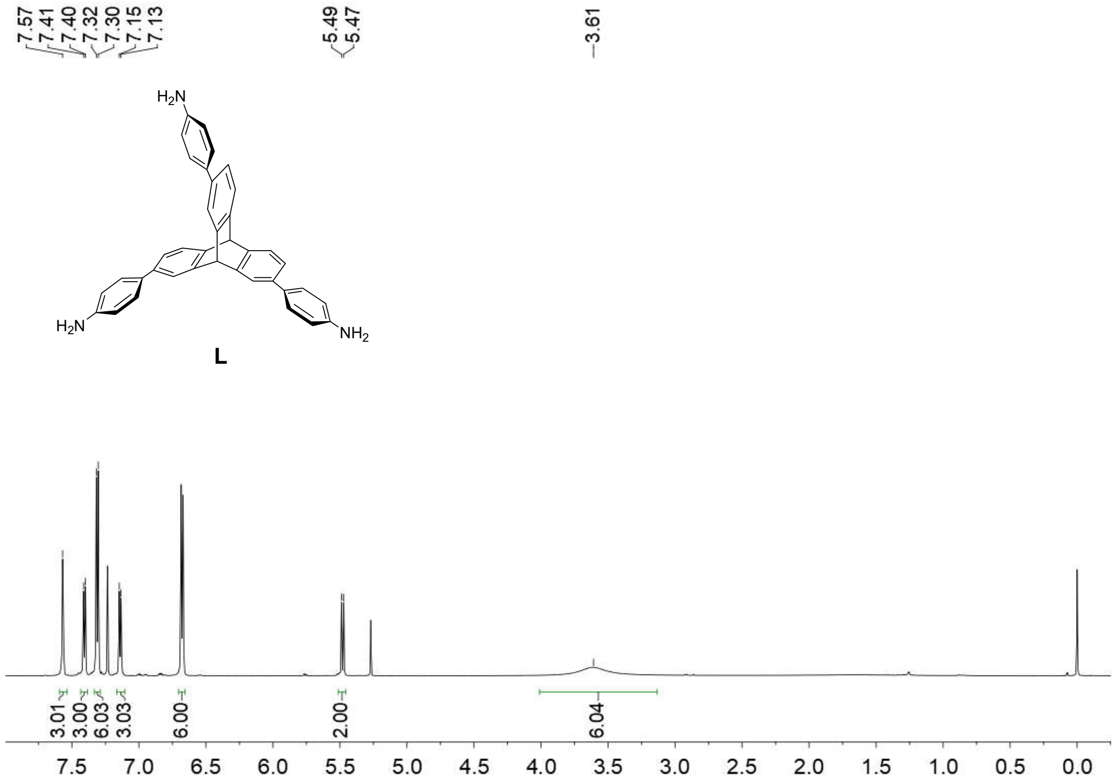

Figure S1. ^1H NMR (600 MHZ, $CDCl_3$) spectrum of **L**.

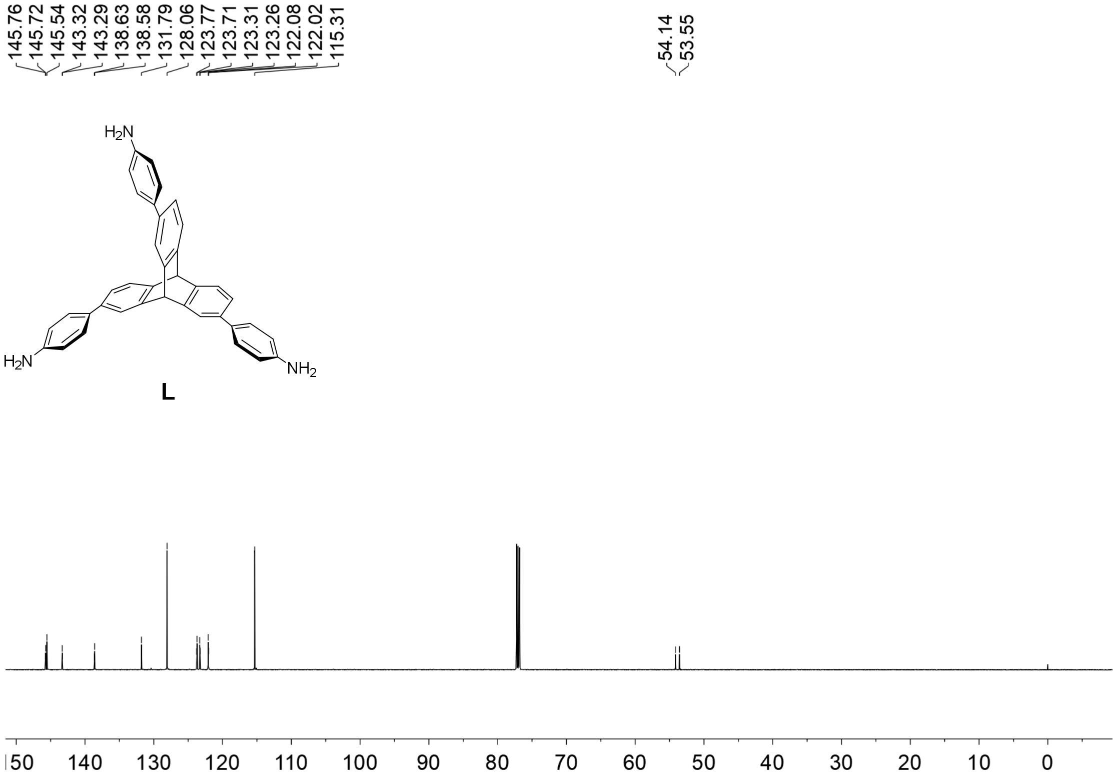

Figure S2. ^{13}C NMR (151 MHZ, $CDCl_3$) spectrum of **L**.

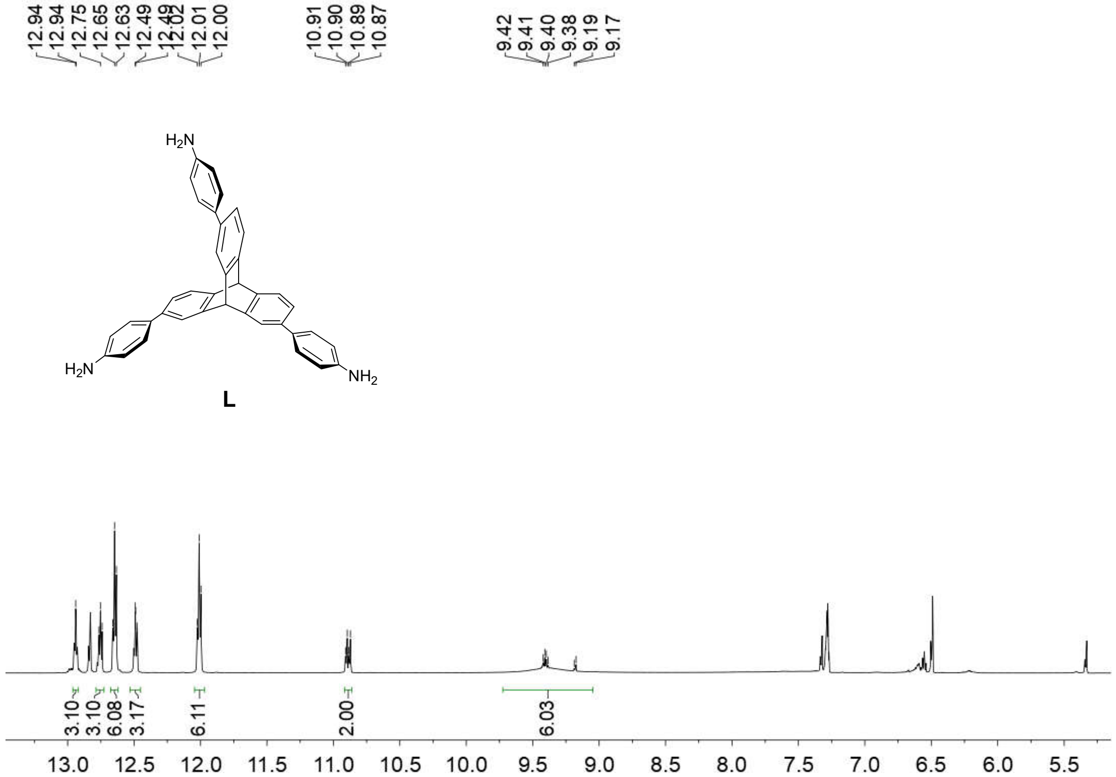

Figure S3. ^{13}C NMR (151 MHZ, $CDCl_3/CD_3CN$ 1:1) spectrum of **L**.

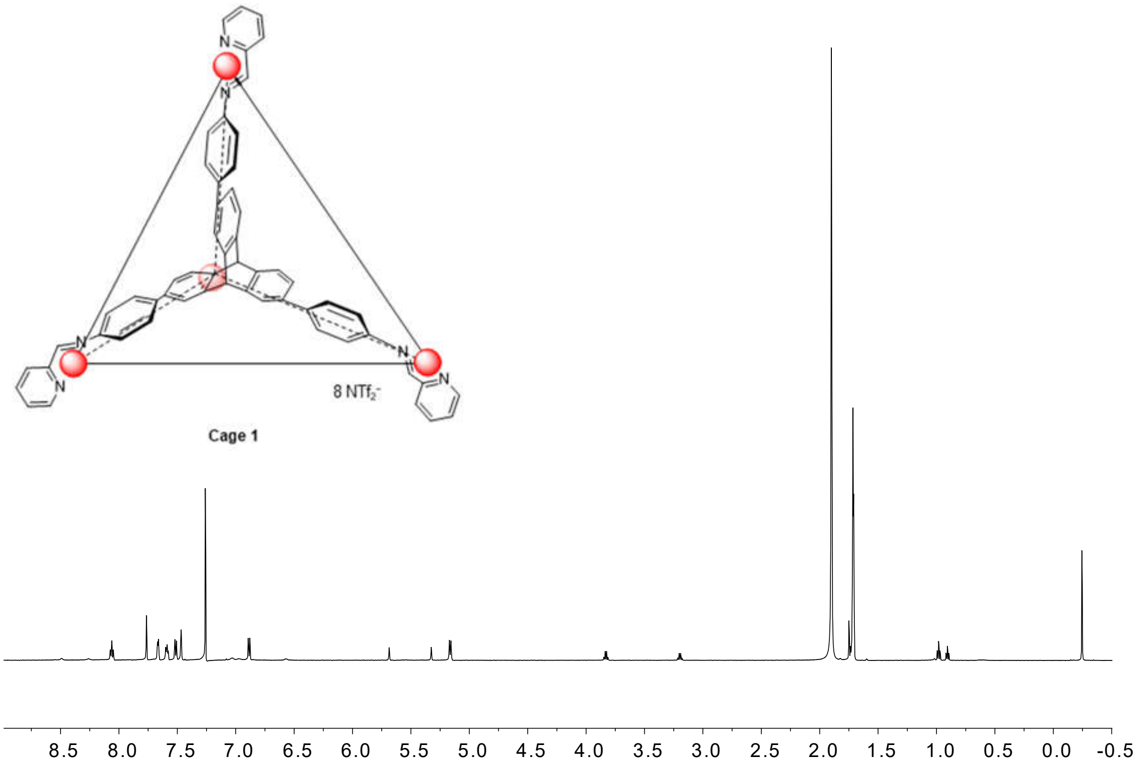

Figure S4. ^{1}H NMR (151 MHZ, $CDCl_3/CD_3CN$ 1:1) spectrum of **Cage 1**.

2. ESI-MS spectrum of Cage 1

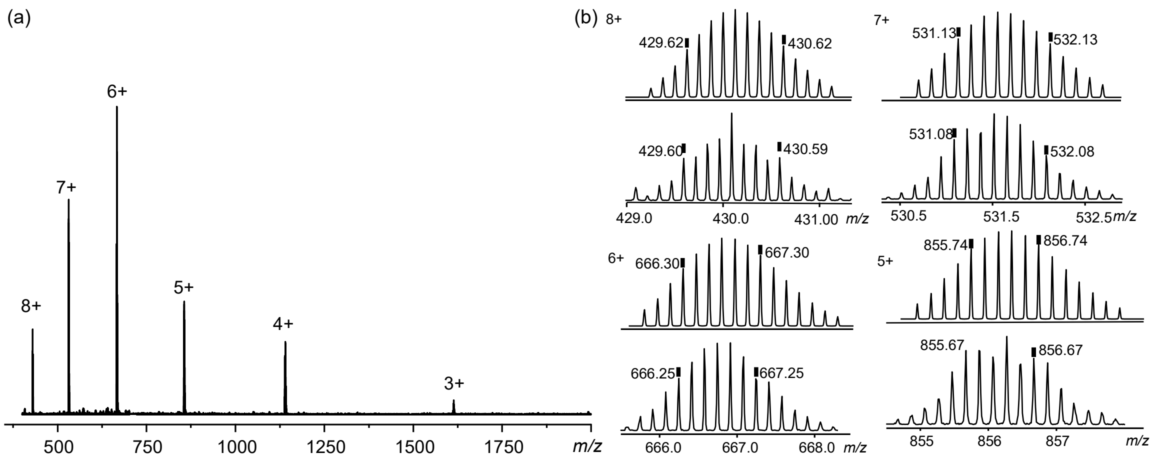

Figure S5. (**a**) and (**b**) ESI-MS spectrum of **Cage 1**. Theoretical (top) and experimental (bottom) isotope patterns for different charge states observed from **Cage 1** (NTf_2^- as the counterion).

3. Generation of the molecular model of Cage 1

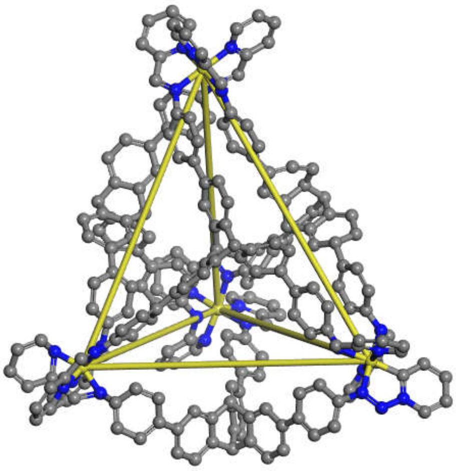

Figure S6. Energy-minimized molecular model of **Cage 1**. (Zn, yellow; N, blue; C, gray). Hydrogens and counter anions are omitted for clarity.

5. Gas adsorption properties of Cage 1

Orange powder **Cage 1** was obtained through direct precipitation of **Cage 1** from its acetonitrile solution after self-assembly by adding diethyl ether. The solid was dried under vacuum overnight. Prior to each measurement below, the sample was treated following the description in Materials and instrumentation [1].

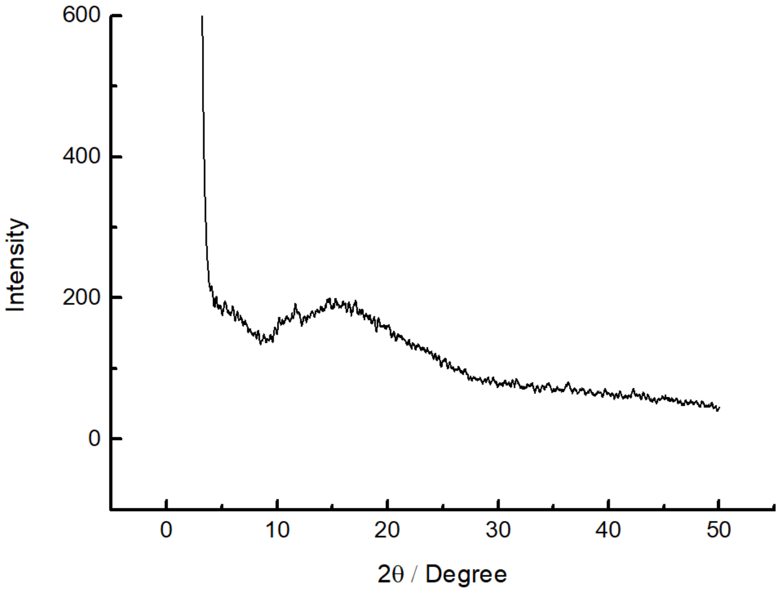

Figure S7. Experimental PXRD pattern of **Cage 1**.

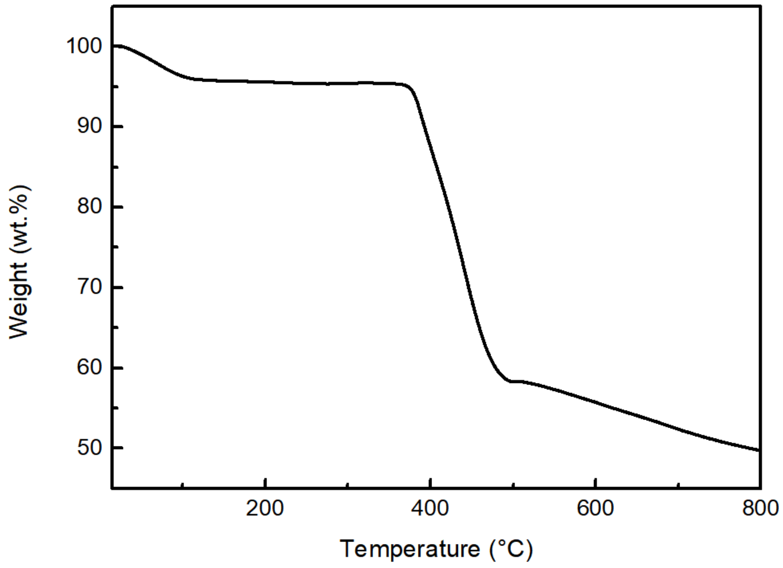

Figure S8. TGA plot of solid **Cage 1** under N_2.

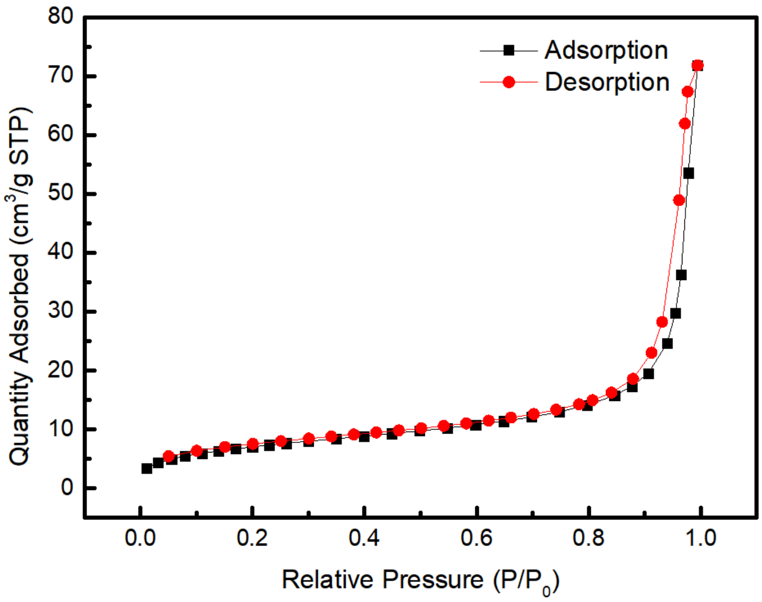

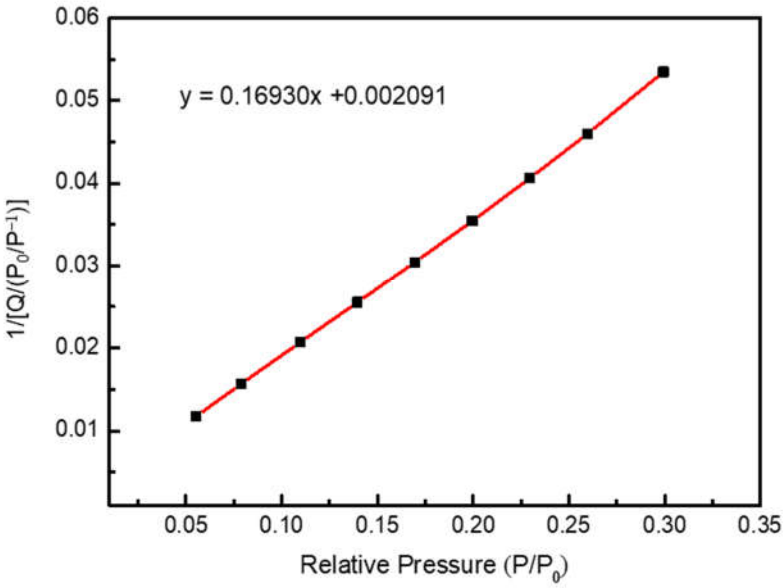

Figure S9. N_2 sorption isotherms of activated **Cage 1** measured at 77 K (upper) and plot of the linear region of the BET equation for **Cage 1** (bottom). $S_{BET} = \frac{VmNA\sigma}{22400W} = \frac{6.022\times10^{23}\times[1/(0.169310+0.002910)]\times1.62\times10^{-17}}{22400} = 25.3955\ m^2/g$

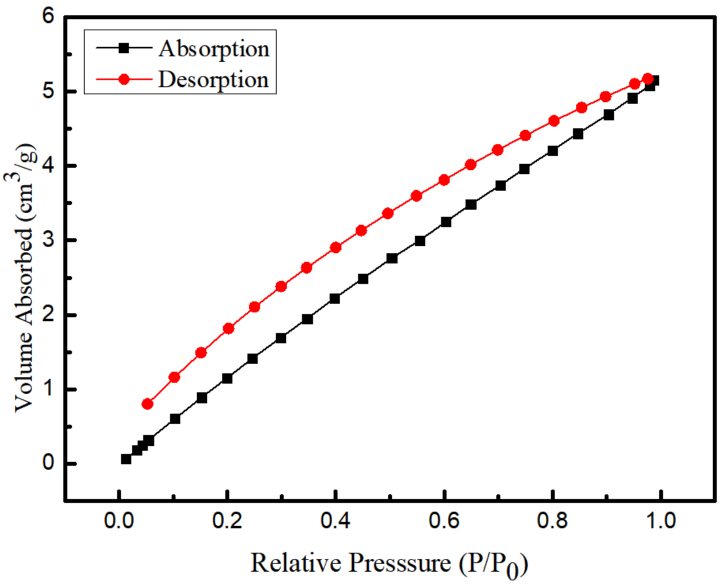

Figure S10. CO_2 adsorption/desorption isotherms of activated **Cage 1** at 298 K.

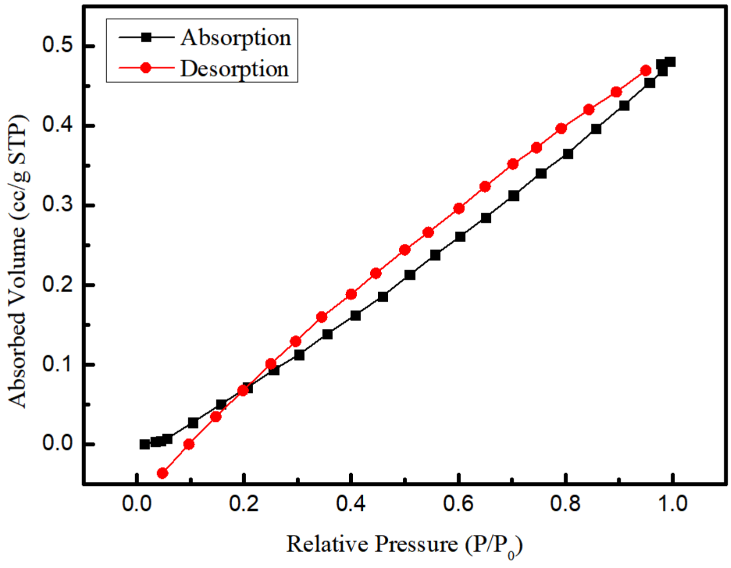

Figure S11. O_2 adsorption/desorption isotherms of activated **Cage 1** at 298 K.

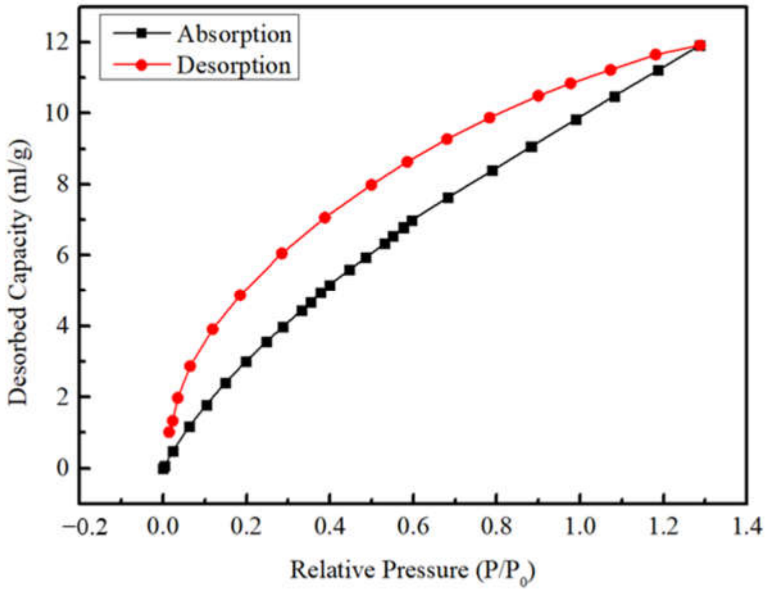

Figure S12. C_2H_2 adsorption/desorption isotherms of activated **Cage 1** at 298 K.

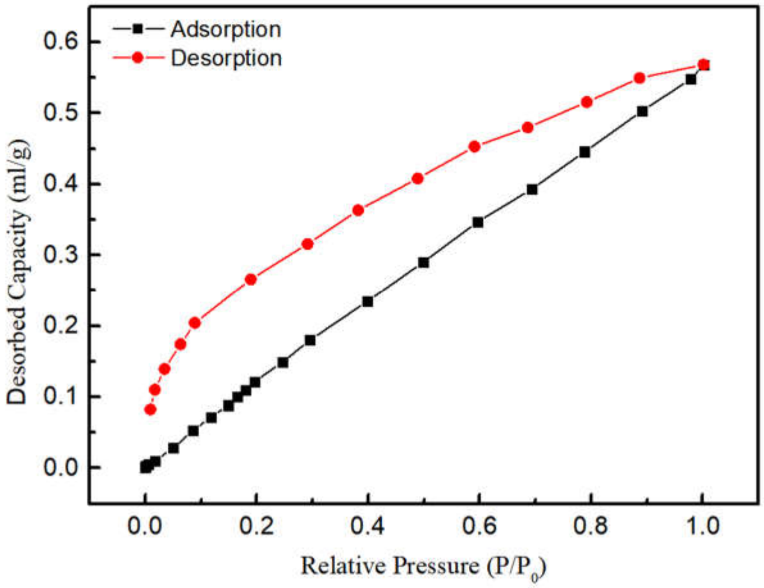

Figure S13. N_2 adsorption/desorption isotherms of activated **Cage 1** at 298 K.

References

1. Zhu, J.L.; Zhang, D.; Ronson, T.K.; Wang, W.; Xu, L.; Yang, H.B.; Nitschke, J.R. A Cavity-Tailored Metal-Organic Cage Entraps Gases Selectively in Solution and the Amorphous Solid State. *Angew. Chem. Int. Ed.* **2021**, *60*, 11789–11792. https://doi.org/10.1002/anie.202102095.
